# High-performance silk/polylactic acid composite scaffold material with immunomodulation and osteogenesis function

**DOI:** 10.1016/j.mtbio.2024.101316

**Published:** 2024-10-28

**Authors:** Jia Rui, Siyu Zhu, Xiang Xu, Yi Wang, Zulan Liu, Guotao Cheng, Dingpei Long, Lan Cheng, Fangyin Dai

**Affiliations:** State Key Laboratory of Resource Insects, Key Laboratory of Sericultural Biology and Genetic Breeding, Ministry of Agriculture and Rural Affairs, College of Sericulture, Textile and Biomass Sciences, Yibin Academy, Southwest University, Chongqing, 400715, China

**Keywords:** Bone regeneration, Flat silkworm cocoon, Polylactic acid, Composite scaffolds, Immunomodulation

## Abstract

The choice of suitable materials and effective structural design are crucial in influencing the therapeutic outcomes of bone tissue engineering scaffolds. This study introduces a controllable biodegradable composite scaffold composed of flat silkworm cocoon (FSC) and polylactic acid (PLA) as an innovative strategy for promoting bone healing in complex injuries. We focused on optimizing the scaffold's structural design, mechanical properties, and underlying mechanisms of osteogenesis. Initial experiments established the parameters for hot pressing the FSC, followed by mechanical performance tests to identify the optimal preparation conditions. Composite scaffolds incorporating PLA films were subsequently fabricated using these optimized parameters. The results indicate that the FSC/PLA composite scaffold exhibits outstanding biocompatibility, mechanical strength, and in vitro mineralization capabilities, alongside an appropriate degradation rate. Furthermore, the composite scaffolds demonstrated significant potential in promoting osteogenic differentiation and facilitating macrophage polarization toward an anti-inflammatory M2 phenotype. In vivo implantation of the scaffold in defective regions enhanced osteogenesis and mitigated inflammatory responses associated with degradation. This investigation presents an optimal composite scaffold that closely mimics the complex structure of bone, offering a novel approach to enhance bone regeneration and effectively address substantial bone defects.

## Graphical abstract

**Figure:**
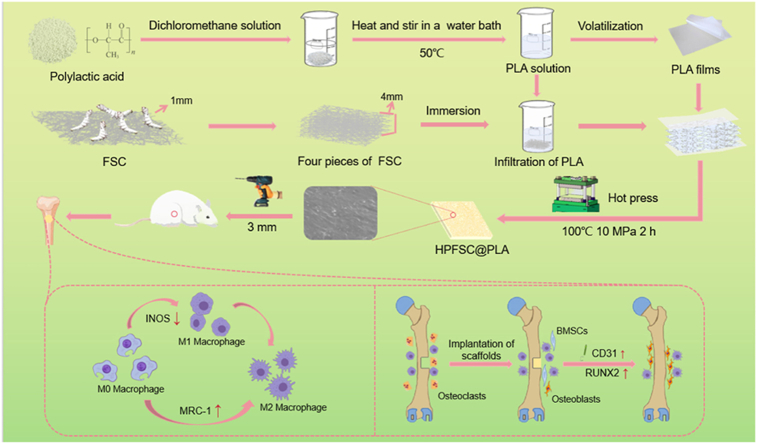

## 1 Introduction

Bones are a type of dynamic hard tissue with high hardness and moderate self-healing ability [1]. The skeletal system in the human body serves multiple crucial functions, including providing a structural framework for muscle and tissue attachment, facilitating bodily movement, and safeguarding organs against potential damage [2]. Bone diseases constitute 50 % of all chronic diseases in individuals aged 50 and above, posing a significant global challenge for clinical treatment [3,4]. Bone defects resulting from trauma, nonunion of fractures, and surgical resection of bone tumors can significantly impact patients' quality of life and give rise to social and psychological challenges [5]. The treatment methods for bone tissue regeneration include autologous bone transplantation, allogeneic bone transplantation, and bone tissue engineering, among which autologous bone transplantation is also known as the clinical gold standard for treating bone defects [6]. However, autologous bone transplantation has drawbacks such as secondary injury, common complications, limited special shape, and limited autologous bone supply, which limit its widespread application in clinical settings [7]. The sources of allografts are diverse, but their biological activity is relatively low, making them susceptible to pathogen infections and immune rejection reactions [[8], [9], [10]]. Therefore, there is an urgent need to develop artificial bone grafts that are worth applying, and bone tissue engineering (BTE) is emerging as a promising alternative for the treatment of bone defects [[11], [12], [13]].

Bone tissue engineering combines stem cells, scaffolds, and growth factors (GFs) to create an optimal biomimetic environment, promoting the regeneration and proliferation of normal tissue cells [14]. Since its proposal in 1995, it has gained significant attention [15,16] and has become a crucial branch in the field of tissue engineering [17]. The selection of an appropriate material for scaffolds is a crucial aspect in the field of bone tissue engineering [18]. Metal grafts, particularly bioinert materials like titanium and its alloys, have emerged as the predominant choice in clinical practice for in-situ repair of bone tissue damage [19,20]. However, the osteogenic induction ability of metal based materials is poor and non-degradable, and the clinical prognosis cannot achieve the expected effect [21]. The development of bone tissue engineering materials possessing excellent biocompatibility, biodegradability, and osteogenic induction is a pressing clinical need.

The critical determinant in tissue engineering scaffolds is the careful selection of biomaterials. Factors such as mechanical strength [22], porosity [23], biocompatibility, and biodegradability [24] significantly impact the biomedical application of the scaffold. It is essential for the scaffold to possess the ability to promote cell adhesion, proliferation, and differentiation in order to facilitate cellular integration into functional tissues. The incorporation of both organic and inorganic materials synergistically enhances the functionality of biomaterials. The three primary categories of biomaterials utilized for scaffold preparation include natural biomaterials, ceramic biomaterials, and synthetic biomaterials [12]. Natural biomaterials have superior biocompatibility and are of great significance for tissue engineering scaffolds [25].

Silk fiber is a natural biopolymer with remarkable mechanical strength, controllable biodegradability, excellent biocompatibility, and ease of processing [[26], [27], [28]]. Various forms of scaffolds, such as electrospinning, hydrogel and 3D printing, have been developed based on regenerated silk fibroin to repair various defects [[29], [30], [31], [32]]. Bianza Moise Bakadia et al. prepared AZM-SS/PVA hydrogels from polyvinyl alcohol (PVA), silk fibroin (SS) and azithromycin (AZM) by repeated freezing and thawing using Genping (GNP) as crosslinking agent [33]. The hydrogel showed sustained release of β and AZM, cell compatibility with keratinocytes and fibroblasts, and skin adhesion during freeze-drying. AZM-SS/PVA hydrogel has better wound healing effect than the commercial Tegaderm membrane dressing, and minimizes the impact on systemic burns. Hao Wang et al. prepared a multifunctional silk fibroin/gelatin-tyramine composite hydrogel catheter by diffusion-driven crosslinking method, with adjustable size and flexibility [34]. They constructed a delivery system of ZIF-8 nanoparticles (miR-29a @ ZIF-8) loaded with miR-29a, and compounded it into SF/GT hydrogel catheter, which improved its biological activity and nerve repair ability through sustained release of MIR-29a. This hydrogel catheter can significantly promote myelination, nerve differentiation and axonal extension of Schwann cells and PC12 cells, and promote the transformation of macrophages from M1 phenotype to M2 phenotype, thus adjusting the immune microenvironment of nerve regeneration, which has potential application value in peripheral nerve repair. Shiyuan Yang et al. mixed dopamine modified silk fibroin (SFD) with chitosan (CS)/β-glycerophosphate (β-GP) to develop an injectable composite hydrogel (SFD/CS/ZIF-8@QCT) containing quercetin modified zeolite imidazole skeleton [35]. This composite hydrogel can quickly stop bleeding after implantation, effectively reduce the reprogramming of Porphyromonas gingivalis and macrophages to M2 polarization, enhance the characteristics of bone/angiogenesis, accelerate the homing of endogenous periodontal ligament stem cells, and finally promote alveolar bone regeneration in periodontitis. The aforementioned studies collectively demonstrate the inherent advantages and immense potential of silk fibroin within the realm of tissue engineering scaffolds. The preparation process of regenerated silk fibroin, however, is intricate and compromises the distinctive fiber structure of silk, leading to a reduction in its mechanical properties and overlooking the potential applications of its unique structure. The flat silkworm cocoon (FSC), which is produced by placing silkworms on a two-dimensional substrate, has attracted much attention from researchers [36]. Due to the controllable dimensions, porous hierarchical fiber network, controllable mechanical properties, and favorable biocompatibility, FSC has been currently demonstrated significant advantages as composite matrices and wound dressing [37,38]. Li et al. successfully prepared a novel antibacterial wound dressing with adjustable size and excellent mechanical properties by grafting FSC with ε-Poly-l-lysine (EPL) using hot pressing technology [39]. Due to its structure similar to ECM, this antibacterial dressing could promote cell proliferation and migration, effectively facilitating wound healing, and was expected to become the next generation of multifunctional bioactive wound dressings. Yi et al. had developed a sunlight-driven rechargeable antibacterial FSC that is grafted with tetracarboxylic dianhydride (BD) and non-metal graphite carbon nitride (g-C3N4) [40]. This FSC exhibited excellent biocompatibility and efficient antibacterial activity, while also addressing the issue of weak bonding between traditional inorganic photosensitive materials and organic polymer carriers. Xu et al. utilized epoxy resin to infiltrate a FSC, successfully fabricating a skin-inspired transparent composite film [37]. This composite thin film exhibited excellent transparency, stretchability, as well as remarkable tear and fatigue resistance, enduring approximately 30,000 loading and unloading cycles before complete rupture. This bio-inspired approach provided a strategy for constructing high-performance functional composite materials. Besides, in comparison to regenerated silk fibroin, FSC can be directly employed, eliminating the laborious process of silk protein dissolution and regeneration.

Polylactic acid (PLA) is a biodegradable hydrophobic polyester that exhibits excellent environmental compatibility and safety for various applications [41,42]. Its compressive strength closely resembles that of natural bone, making it an ideal material for bone tissue regeneration [43]. The degradation products of PLA have been extensively studied and proven to possess exceptional biocompatibility and low cytotoxicity in local tissues during the process of bone tissue regeneration. Due to its remarkable thermal processability, biocompatibility, and degradability, PLA finds widespread utilization in the fabrication of materials for regenerating bone tissues [44].

The scarcity of suitable materials that provide the required mechanical strength, biocompatibility and degradation rate while promoting osteogenic differentiation, as well as the inability of conventional scaffolds to modulate inflammatory responses are the critical challenges for bone regeneration in clinical application. PLA is a biodegradable polymer with strong mechanical properties, which enhances the structural integrity provided by flat silkworm cocoon (FSC) in bone tissue repair. Its biocompatibility and widespread medical use make it an ideal choice, promoting tissue integration and supporting new bone growth. PLA's degradation profile, aligned with that of FSC, allows for a controlled degradation process, ensuring support throughout bone regeneration. FSC, on the other hand, serves as a natural biomimetic matrix, facilitating cell adhesion, proliferation, and osteogenesis. Its unique structure mimics the extracellular matrix and enhances immunomodulation, helping to reduce inflammation during healing.

**Fig. 1:**
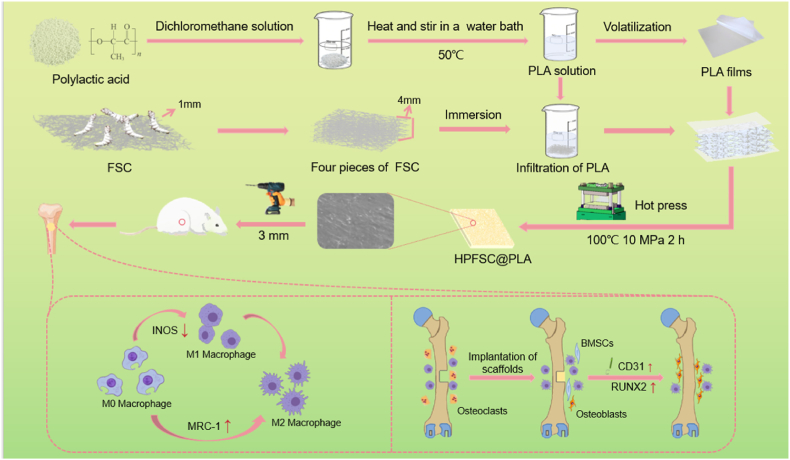
The schematic representation and fabrication process of an FSC/PLA implantable stent designed for femoral defect repair.

In this study, we aim to tackle these challenges by developing a novel composite scaffold made from FSC and PLA that not only enhances mechanical properties and biocompatibility but also possesses immunomodulatory effects to facilitate osteogenesis, as illustrated in Fig. 1. A comprehensive physicochemical analysis of the fabricated scaffold were firstly conducted, and the impact of the scaffold on promoting proliferation, differentiation, and mineralization of MC3T3-E1 osteoblasts was assessed. Additionally, the immunomodulatory effects of HPFSC@PLA scaffolds on macrophage (RAW264.7) polarization were systematically evaluated. Finally, the efficacy of HPFSC@PLA composite scaffolds in bone regeneration was evaluated using a rat femoral defect model. The HPFSC@PLA composite scaffold exhibited enhanced osteogenic potential and anti-inflammatory capabilities, positioning it as a promising candidate material for bone repair.

## 2 Materials and methods

### 2.1 Materials

The silkworm species (National Silkworm Genetic Resources Gene Bank, Chongqing, China) were carefully selected and reared until they reached the fifth instar stage. A specific number of silkworms (approximately 5 kg/m^2^) were then confined on a two-dimensional plate after defecation to obtain FSC with a thickness of 1 mm. PLA particles and dichloromethane solution were obtained from Shanghai Macklin Biochemical Co., Ltd. (China). All reagents were analytically grade.

### 2.2 Fabrication of PLA film

The PLA particles weighing 20 g were carefully measured and transferred into a beaker. Subsequently, a dichloromethane solution of 400 g was added to the beaker, which was then sealed with cling film. The beaker was positioned in a water bath at a constant temperature of 50 °C and continuously stirred until complete dissolution of the PLA particles occurred. Following this, the resulting PLA solution was poured into a square concave mold and evenly distributed. After an evaporation period of 12 h, a well-formed PLA film was obtained and collected for further use. A uniform square-shaped PLA film measuring 10 cm × 10 cm was precisely cut for subsequent experiments.

### 2.3 Fabrication of FSC/PLA composite scaffold

The FSC was cut into a 10 cm × 10 cm square shape, and a flat vulcanization machine was utilized to fabricate composite scaffolds through hot pressing based on the layering method involving one layer of P (PLA film) and one layer of F (FSC film). The stacking approach comprising three PLA films between four FSC sheets was denoted as HPFSC/3PLA. Similarly, the stacking technique involving four FSC pieces with three layers of PLA film in between, and covering each side with one piece of PLA film was referred to as HPFSC/5PLA. In the case of HPFSC@PLA, four pieces of flat silkworm cocoon were soaked in PLA solution, covered on both sides with PLA film, and subsequently subjected to hot pressing. Lastly, direct heat pressing without any additional layers or treatments for the four pieces of flat silkworm cocoon was termed as HPFSC. *The FSC:PLA ratio in HPFSC/3PLA is 1: 3, the FSC:PLA ratio in HPFSC/5PLA is 1: 5, and that in HPFSC@PLA is 1: 7.*

### 2.4 Characterization

The surface morphology of the materials was characterized using scanning electron microscopy (SEM, Hitachi, SU3500, Japan). A thin layer of gold was applied to coat the sample surface, and images were collected under an accelerated voltage of 15 kV. Tensile strength tests on hot pressed FSC and composite brackets were conducted using a universal electronic testing machine (model: E44.104, MTS System (China) Co., Ltd.) at a stretching speed of 2 mm/min. The strip sample had dimensions of 60 mm in length, 10 mm in width, and a stretching spacing of 10 mm. The stress-strain curve of the sample was recorded. Fourier transform infrared spectroscopy (FTIR) with a Nicolet Is10 (Thermo Scientific) spectrometer frontier was employed to detect the protein structure of the materials within a spectral region ranging from 4000 to 800 cm^−1^. X-ray diffraction (XRD) analysis using Cu-Kα as radiation source was performed on the materials with a Rigaku Ultima IV instrument from Japan. The scanning range for XRD analysis spanned from 2θ = 10–80° at a scanning speed of 5°/min.

### 2.5 In vitro degradation assay

The composite scaffolds were subjected to in vitro degradation by incubating them in PBS solution at 37 °C and 5U protease XIV PBS solution, respectively. Each scaffold was incubated with 1 mL of the respective solution. The incubation solutions were changed every two days. At designated time points (3 d, 7 d, 15 d, 30 d, 60 d), the samples were rinsed with deionized water and dried with natural wind before being weighed. The remaining mass of each sample was recorded and SEM images of the samples were captured.

### 2.6 In vitro mineralization assay

The composite scaffolds were subjected to in vitro mineralization by incubating them in 1 mL of SBF (simulated body fluid) at 37 °C. The SBF solution was replenished every two days. At designated time points (3 d, 7 d, and 14 d), the scaffolds were lightly washed with deionized water to remove excess SBF solution and air-dried naturally. Subsequently, SEM images were captured after complete drying of the samples.

### 2.7 Cytocompatibility evaluation

#### 2.7.1 Cell culture and seeding

The MC3T3-E1 mouse embryonic osteoblast precursor cells used in this study were obtained from the Cell Repository of the Typical Culture Collection Committee of the Chinese Academy of Sciences/Shanghai Institutes of Life Sciences Cell Resource Center of the Chinese Academy of Sciences. The cells were cultured in DMEM medium supplemented with 10 % (w/v) fetal bovine serum (FBS; Gibco, Australia) and 1 % (10,000 U/mL) penicillin/streptomycin (Gibco, U.K.) at 37 °C and 5 % CO_2_, with medium changes every two days. Upon reaching confluence, cells were detached using 0.25 % Trypsin-EDTA (Gibco), centrifuged, and resuspended for seeding onto scaffolds that had been sterilized with 70 % ethanol, washed twice with sterile PBS, and irradiated by short-wavelength UV-light for 2 h.

#### 2.7.2 Cell proliferation assay

The MC3T3-E1 cells were seeded onto the scaffolds at a density of 2 × 10^4^ cells/ml in 48-well plates. Cell proliferation was evaluated using the MTS assay after co-culturing with the scaffolds for 24 h and 48 h, respectively. At the designated time points (24 h and 48 h), the culture medium was aspirated and replaced with 500 μL of PBS solution to carefully wash the cells once. Subsequently, each well was treated with 500 μL of MTS working fluid (MTS: DMEM = 1:5) and incubated in darkness for 1 h within a cell culture incubator. Finally, an aliquot of 100 μL from each well was transferred to a separate well in a 96-well plate, and absorbance at 490 nm was measured using a microplate reader (Synergy H1, Biotek). Cells cultured in DMEM medium containing10 % FBS served as controls, and their absorbance values were used to determine cell viability as percentage survival rate. The results were expressed as cell viability:

cellviability(%)=(Ae‐An)/(Ap‐An)×100%,

where Ap was the absorbance of the positive control group, An was the absorbance of the negative control group, and Ae was the absorbance of the experimental group.

#### 2.7.3 Live/dead staining

According to the aforementioned methodology, cells were seeded onto the scaffold. Following a 24-h incubation period, the sample was subjected to LIVE/DEAD staining. The staining solution, composed of 30 μL of 1.5 mM propidium iodide (US Everbright) and 5 μL of 4 mM Calcein AM (US Everbright), was prepared in PBS solution. Subsequently, each sample received an addition of 100 μL staining solution and was incubated in darkness at a temperature of 37 °C for a duration of 30 min. A super-resolution confocal microscope (Olympus, BX3-SSU, Japan) was employed for observation purposes. Live cells exhibited green fluorescence while dead cells displayed red fluorescence upon staining. Image J software facilitated quantitative analysis pertaining to live and dead cell populations.

#### 2.7.4 Cell adhesion and morphology assay

After 48 h of co-cultivation, the original culture medium was removed and the sample was cleaned three times with PBS. Subsequently, the cells were fixed with 4 % paraformaldehyde for 20 min and washed three times with PBS. Then the cells were permeated with 0.1 % Triton X-100 at room temperature for 15 min. The Triton X-100 solution was removed, and the cells were washed three times with PBS. Next, the cells were sealed with 1 % BSA solution for 1 h. Added FITC labeled ghost pen cyclic peptide in the dark, incubated at 37 °C for 1 h, and washed three times with PBS. Finally, DAPI was added in dark to stain the cell nucleus for 5 min and the cells were washed three times with PBS. A super-resolution laser confocal fluorescence microscope (Olympus, BX3-SSU, Japan) was used to observe the adhesion of MC3T3-E1 on the scaffold material after staining the sample.

### 2.8 Hemolysis assay

The hemolytic activity method was used to determine the blood compatibility of the composite stent. Universal centrifugation (1000 rpm, 12 min) was used to obtain red blood cells from rat blood. Purified red blood cells were washed 5 times with PBS solution, and the supernatant did not show any red color. Then 40 μL purified red blood cells were added to 1960 μL diluted the red blood cell suspension to a final concentration of 2 % (v/v) in PBS solution, and 1 mg of scaffold samples was add to each group. The RBC suspension containing 1 % (v/v) Triton X-100 was used as the positive control group, while the PBS treatment group was used as the negative control group. The prepared suspension of each group was incubated at 37 °C for 2 h, and centrifuged at 2500 rpm for 5 min after incubation. Then, 100 μL the supernatant was transferred to a new 96-well plate. A microplate reader was used to measure the absorbance of the solution at a wavelength of 540 nm. The results were expressed as hemolysis rate:

Hemolysis%=(Dt‐Dnc)/(Dpc‐Dnc)×100%,

where Dt, Dpc, and Dnc were the absorbance of the sample, positive, and negative control, respectively.

### 2.9 ALP assay

The MC3T3-E1 cells were seeded in 6-well plates with a density of 1 × 10^5^ cells/mL. When the cells grew to 70–80 % fusion, the culture medium was replaced with osteogenic induction medium (DMEM complete culture medium supplemented with 50 μg/mL ascorbic acid, 10 mM *β*-Phosphate glycerol, 100 nM dexamethasone), and each group of scaffold materials was immersed in the culture medium. The group without scaffold materials was used as a control. The osteogenic induction medium was changed every two days. On the 7 and 14 d, the cell plates were removed and the culture medium was discarded, the cells were rinsed with PBS three times, and fixed in 4 % paraformaldehyde for 10 min. Then, the instructions were followed to stain with BCIP/NBT reagent kit (Beyotime) for 30 min. An inverted optical microscope was used to observe the stained cells and take photos. Image J was used to quantify the generated alkaline phosphatase.

### 2.10 ARS staining and quantification

The method was the same as above. On the 14th day, the cell plate was removed, and 1 mL of fixative was added to fix the cells for 20 min. Then the cells were washed three times with PBS and 1 mL of ARS staining solution was added. The cells were stained at room temperature for 30 min and washed three times with DI H_2_O to remove the unattached ARS. An inverted optical microscope was used to observe the stained cells and take photos. Image J was used to quantify the generated ARS.

### 2.11 Macrophage polarization assay

When RAW267.4 was cultured in a culture bottle until 70–80 % fusion occurs, the cells were seeded with a density of 5 × 10^4^ cells/mL into a twelve well plate, and cultured for 12 h. Then the medium was removed and the cells were washed three times with sterile PBS. 500 μL 0.5 μg/mL lipopolysaccharide LPS (prepared from DMEM basic culture medium) was add to each well to induce for 12 h. 1 mL of DMEM complete medium was added and the scaffold material was placed in to incubate for 48 h. At room temperature, the cells were fixed with 500 μL 4 % paraformaldehyde for 10 min and washed three times with PBS. 500 μL 0.1 % Trixton X-100 were added to each well and the cells were penetrated at room temperature for 10 min. The penetrating agent was removed and the cells were cleaned with PBS three times, each time for 5 min. 1 mL 1 % BSA solution was added to each well and the cells was incubated at room temperature for 1 h. 500 μL diluted primary antibody (M1 type primary antibody is INOS, M2 type primary antibody is MRC-1) was added to each well, and the cells were incubated overnight at 4 °C. 500 μL diluted secondary antibody (dissolved in PBS containing 1 % BSA) was added to each well and the cells were incubated at 37 °C in the dark for 2 h. 500 μL DAPI staining solution was added to each well, the nucleus were stained for 10 min and washed three times with PBS. A super-resolution confocal microscope (Olympus; BX3-SSU, Japan) was used to observe the fluorescence intensity.

### 2.12 Femoral defection model in rats

The animal experiment was approved by the Ethics Committee of Experimental Animals at Southwest University, and the experimental procedures were carried out in accordance with the guidelines of the China Association for the Management of Experimental Animals on the care and use of experimental animals. All Sprague Dawley (SD) rats were male at eight weeks of age and had been adapted for one week before undergoing surgery. Ten SD rats (2 time points) were anesthetized by intramuscular injection of Zoletil 50 (Virbac). After anesthesia, the rats were fixed on the operating table and the skin around the femur was cleaned with disinfectant. Then make an incision about 2–3 cm long in the middle part of the femur, and carefully expose the femur. Then, a hole with a diameter of 3 mm and a depth of 3 mm was drilled at the incision perpendicular to the femur of the rat's legs with a drill bit, and the material was filled into the drilled hole in the direction parallel to the femur. The left leg femur defect was used as a blank control, and a composite stent was implanted at the right leg femur defect. Finally, the skin was sutured and penicillin was injected. Two time points (3 w, 6 w) was set, with five repetitions for each time point. At the time point, the corresponding rats was euthanized. Femurs and the five internal organs were collected and soaked in a 4 % paraformaldehyde solution for subsequent testing.

### 2.13 Micro-CT analysis

After euthanasia of rats, the femurs were collected and fixed. The sample was scanned with a miniature CT scanner to obtain 2D images, 3D reconstructed images, as well as parameters including bone volume fraction (BV/TV), bone mineral density (BMD), and bone trabecular separation (Tb.sp).

### 2.14 Histological analysis

After scanning with Mirco-CT, the sample was cleaned with ddH_2_O, decalcified with 10 % EDTA at room temperature for one month, dehydrated with ethanol gradient, embed in paraffin, and cut into 5 μm wax slices. Bone tissue was stained with Masson's trichrome and hematoxylin eosin (H&E). Immunohistochemical OCN and RUNX2 fluorescence staining were used to evaluate new bone formation. Immunomodulatory effects were evaluated by immunofluorescence double staining of INOS and MRC-1. The images stained with Masson's trichrome and hematoxylin eosin (H&E) were collected using fluorescence microscopy. The images stained with immunohistochemistry were collected using a super-resolution confocal microscope.

At the 6th week after surgery, the rats were euthanized and their hearts, liver, spleen, lungs, and kidneys were removed and fixed in 4 % paraformaldehyde. Samples were dehydrated with ethanol gradient, embed in paraffin, and cut into 5 μm wax slices. Subsequently, hematoxylin eosin (H&E) staining was performed on the sample. The images stained with hematoxylin eosin (H&E) were collected using a fluorescence microscope.

### 2.15 Statistical analysis

All tests were performed at least three times and all final calculated data were expressed as mean ± standard deviation (SD). Origin software was used to analyze all the data and ∗p < 0.05 was considered statistically significant.

## 3 Results and discussion

### 3.1 Fabrication and characterization of the hot-pressed FSC

Hot pressing is a commonly used method in fabricating composite materials. In this study, we initially subjected FSC specimens to hot pressing under different conditions of temperature, time duration, and pressure to examine their effects on modifying the properties of FSC. Following that, we increased the number of FSC layers during hot pressing and established optimal parameters through extensive testing for preparing FSC/PLA composite scaffolds subsequently. Utilizing these optimized parameters as guidelines, we successfully fabricated composite scaffolds for further investigation.

**Fig. 2:**
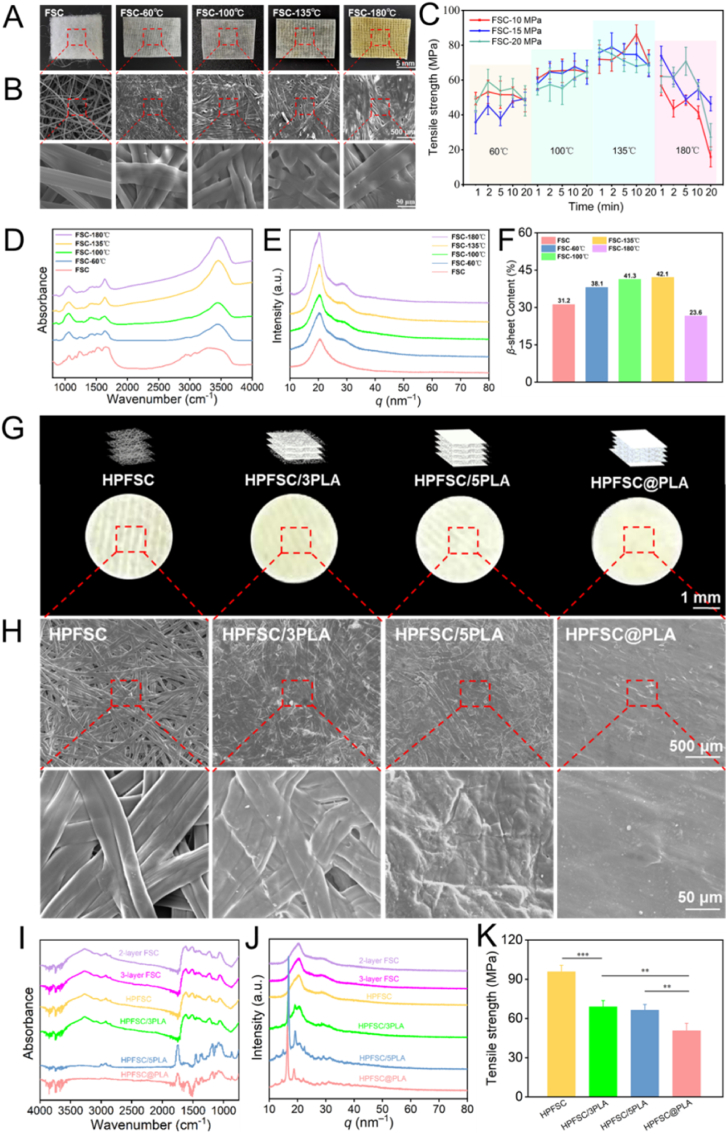
Photographs (A) and SEM (B) of FSC hot pressed at different temperatures; (C) The tensile strength of FSC under different hot pressing parameters; (D, E) FTIR and XRD of FSC hot pressed at 10 MPa, 10 min and different temperatures; (F) The *β*-sheet content of FSC and FSC hot pressed at 10 MPa, 10 min and different temperatures; Photographs (G) and SEM (H) of HPFSC and FSC/PLA composites; (I, J) FTIR and XRD of different layers of hot pressed FSC and HPFSC/PLA composite scaffolds; (K) The tensile strength of HPFSC and HPFSC/PLA composite scaffolds.

The photographs and SEM images (Fig. 2A and B) illustrate that the fiber structure of FSC gradually undergoes densification as the temperature increases under constant hot-pressing time and pressure conditions. With increasing temperature, sericin melts and combines with silk fibroin under pressure to form a compact fiber network. The mechanical properties of hot-pressed FSC (Fig. 2C) were tested under different parameters, revealing that the tensile strength of FSC is more significantly influenced by hot pressing temperature than time and pressure. At a hot pressing temperature of 135 °C, FSC exhibits a maximum tensile strength of 84.1 MPa when subjected to corresponding pressure and time conditions of 10 MPa and 10 min, respectively. Moreover, an increase in hot pressing temperature induces changes in the protein structure of FSC, affecting *β*-Sheet impact on the mechanical properties of silk protein as demonstrated by FTIR analysis (Fig. 2D). The absorption peak at the amide I band (1700-1600 cm^−1^) gradually intensifies with rising temperatures up to 135 °C, indicating an increased *β*-sheet content in FSC (Fig. 2F). The XRD results (Fig. 2E) indicate no significant alterations in the crystal structure of silk; however, at a hot pressing temperature reaching180 °C denaturation occurs in silk protein leading to a substantial decrease in its mechanical properties.

The FSC samples underwent layer-by-layer hot pressing at a constant pressure of 10 MPa, while the hot-pressing temperature and duration were varied to investigate their effects on the hot pressing process and mechanical properties of 2–5 layers of FSC (Fig. S1). It was observed that lower temperatures during hot pressing were insufficient for shaping FSC, whereas higher temperatures resulted in denaturation on the surface of FSC, leading to a significant decline in mechanical properties. Tensile strength data revealed that the maximum strength of 4-layered FSC after hot pressing was comparable to that of 3-layered FSC, while providing increased thickness. Considering all factors, including these findings, it was determined that using 4 layers of FSC would be optimal for preparing FSC/PLA composite scaffolds via hot pressing. The selected parameters for this process were fixed at 100 °C, 10 MPa, and a duration of 2 h.

### 3.2 Fabrication and characterization of the FSC/PLA composite scaffold

The photographs and SEM images (Fig. 2G and H) depict the surface morphology of three types of FSC/PLA composite scaffolds and HPFSC. It is evident that the fiber network of HPFSC closely resembles that of a single-layer FSC. The surface fiber structure of HPFSC/3PLA remains distinctly visible. At elevated temperatures, the internal PLA film melts and fills the pores within the inner FSC, while only a small amount of PLA is present in the fiber gaps on the surface FSC. Both HPFSC/5PLA and HPFSC@PLA are coated with a PLA film on their surfaces. During hot pressing, this PLA film melts and fully infiltrates into the fiber gaps on the surface FSC, ultimately completely enveloping the scaffold. The results obtained from FTIR and XRD analyses (Fig. 2I and J) further confirm the presence of PLA on both HPFSC/3PLA and HPFSC/5PLA surfaces, providing evidence for successful preparation of HPFSC/PLA composite scaffolds. In terms of XRD results, characteristic peaks corresponding to α-crystal structure of PLA exhibit a decreasing trend at 2θ = 16.8° and 18.9° [45,46], while the characteristic peaks of silk also showed a decreasing trend around 2θ = 20°. The decrease in crystallinity of the composite scaffold and the increase in interfacial compatibility were evident. Following hot pressing, the tensile strength of PLA and FSC composites decreased due to the relatively low strength of PLA, with this decline being observed alongside an increase in PLA content (Fig. 2K). Nevertheless, HPFSC@PLA still exhibited a commendable final strength of 50.61 MPa. Additionally, at a load of 50 N (Fig. S2), it achieved a compressive stress of 65 MPa, which falls within the typical compressive stress range for cancellous bone (0.2–80 MPa) [47].

### 3.3 In vitro degradation of the scaffolds

**Fig. 3:**
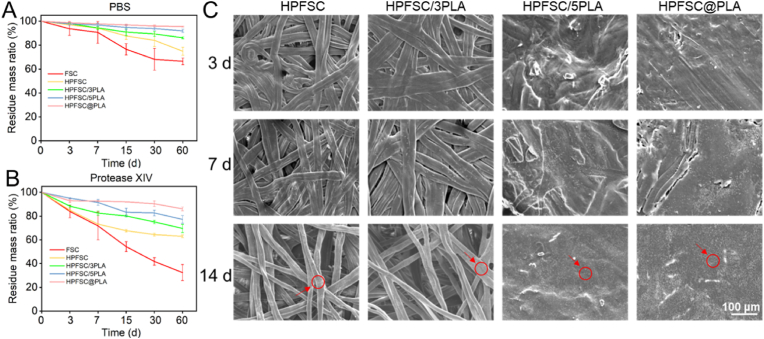
In vitro degradation profiles of FSC, HPFSC and composite scaffolds in PBS solution (A) and protease XIV solution (B); Mineralization of HPFSC and FSC/PLA composites in vitro (C).

The degradation rate of scaffold materials should ideally be synchronized with the growth rate of new bone. Excessive rapid degradation may surpass the growth rate of new bone. The femur's growth rate is typically characterized by a slow pace, with a repair duration that can extend up to 3–5 months. Therefore, it is crucial to ensure that the degradation time of the stent used for femoral repair aligns with this specific timeframe. In this study, we evaluated the biodegradability of composite material scaffolds and presented the results in Fig. 3(A and B). The degradation rate of composite scaffolds in PBS solution and protease solution was slower compared to FSC and HPFSC; however, HPFSC@PLA still maintained 80 % residual mass in protease XIV PBS solution after 60 days due to its higher PLA content. Notably, PLA on the surface of the scaffold exhibited initial degradation in both PBS solution and protease solution at a slower rate than silk protein, effectively reducing overall scaffold degradation as observed in SEM images (Fig. S3). Polymer material degradation initiates from its surface. In groups such as HPFSC/5PLA and HPFSC@PLA, silk is encapsulated and covered by an outer layer of PLA which degrades first over time through hydrolysis on its surface before exposing inner silk and initiating further degradation. PLA degrades more slowly than FSC, providing prolonged mechanical support during the initial stages of bone healing. In contrast, FSC degrades more rapidly, facilitating cell adhesion and promoting early-stage bone regeneration. Therefore, the HPFSC/PLA composite scaffold exhibits a partially degradable characteristic, as its different components degrade at distinct rates, which is crucial for matching the scaffold's degradation with new tissue formation.

### 3.4 In vitro mineralization of scaffolds

When assessing the mineralization performance of composite scaffolds in vitro, simulated body fluid (SBF) was employed to mimic the physiological environment of the human body, while scanning electron microscopy (SEM) was utilized to examine the surface morphology of the scaffold at corresponding time intervals. As depicted in Fig. 3C, minimal amounts of white particles were observed on each scaffold's surface after 3 days, with HPFSC and HPFSC/3PLA exhibiting almost no particle deposition. Following incubation in SBF for 7 and 14 days, increased quantities of white particles were detected on the surfaces of HPFSC/5PLA and HPFSC@PLA compared to the other two groups. Silk itself possessed osteoinductive properties that facilitated hydroxyapatite formation. The addition of PLA enhanced surface reactivity and mimicked a bone membrane due to its thin layer structure, thereby further augmenting mineralization capability. These findings suggest that hot pressing-based combination of PLA and FSC is more favorable for bone-like hydroxyapatite formation.

### 3.5 The cytocompatibility and hemocompatibility of scaffolds

**Fig. 4:**
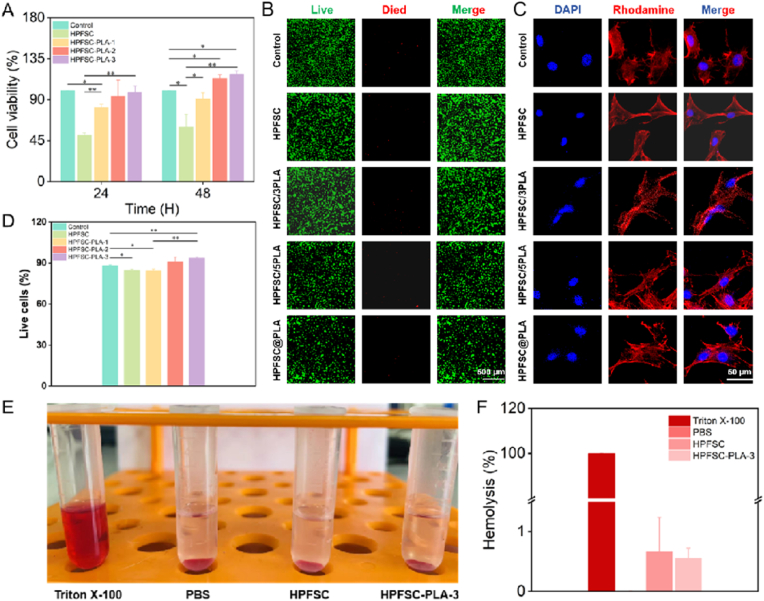
(A) Cell viability results of MC3T3-E1 cells; (B) Live/Dead staining of MC3T3-E1 after 48 h of co-cultivation; (C) Adhesion of MCT3T3-E1 cells on various scaffolds; (D) Quantitative analysis of Live/Dead staining.

MC3T3-E1 cells were co-cultured on composite scaffolds, and their biocompatibility was assessed in vitro using MTS assays and Live/Dead staining. The MTS assay evaluated the proliferation of MC3T3-E1 cells after 24 and 48 h of co-culture with the scaffolds (Fig. 4A). After 48 h, all cell groups showed proliferation. Compared to the control group, the HPFSC group exhibited significant cytotoxicity. However, the cytotoxicity decreased when the scaffold was combined with PLA. Notably, HPFSC/5PLA and HPFSC@PLA significantly promoted cell proliferation. Live/Dead staining observed through laser confocal microscopy (Fig. 4B) revealed fewer dead cells in the HPFSC/5PLA and HPFSC@PLA scaffolds compared to the other groups. Quantitative results (Fig. 4D) indicated that the number of live cells in these two groups reached 90 %, confirming that FSC/PLA composite scaffolds exhibit reduced cytotoxicity. Further research indicated that the combination of sericin and fibroin can induce an immune response, leading to cellular toxicity and inflammation. The introduction of PLA significantly improved the biocompatibility of hot-pressed FSC by enhancing cell activity. As shown in Fig. 4C, cells co-cultured with the scaffold for 48 h exhibited extended pseudopodia compared to the blank control group, indicating better cell adhesion and a more suitable environment for cell attachment and growth. The smooth surfaces of HPFSC and HPFSC/3PLA limited cell growth, while HPFSC/5PLA and HPFSC@PLA promoted cell adhesion and growth. This improvement is likely due to the melting of PLA under high-temperature conditions during the preparation of hot-pressed composite materials, forming an adhesive coating on the FSC surface that enhances cell adhesion and growth.

The scaffolds need to be implanted into the body for repairing bone tissue defects. Therefore, this study evaluated the hemolysis of FSC/PLA composite scaffolds to assess their blood compatibility. As shown in Fig. S3, red blood cells treated with HPFSC and HPFSC@PLA did not exhibit significant hemolysis. Calculations presented in Fig. S4 revealed that the hemolysis rates for these groups were 0.66 % and 0.55 %, respectively, both of which are well below the 5 % threshold, meeting the prescribed hemolysis standards.

### 3.6 FSC/PLA composite scaffold enhance osteogenic differentiation

**Fig. 5:**
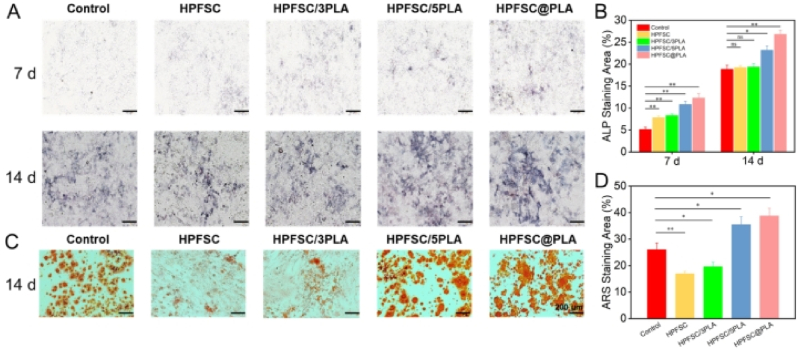
(A) ALP staining of MC3T3-E1 cells cultured for 7, 14 days; (B) Quantitative analysis of ALP; (C) ARS staining of MC3T3-E1 cells cultured for 14 days; (D) Quantitative analysis of ARS.

The ALP enzyme is indispensable for osteogenesis, being secreted by osteoblasts and serving as an early marker of osteogenic differentiation. It plays a pivotal role in the process of mineralization [48,49]. This study investigated the effect of composite scaffolds on MC3T3-E1 osteogenic differentiation through ALP staining and quantitative analysis, while ARS staining and quantitative analysis were used to analyze calcium nodule formation. As shown in Fig. 5A, after 7 and 14 days of cultivation on the scaffold, the HPFSC@PLA group exhibited the highest ALP activity, with more intense staining compared to the other groups. At 14 days, increased staining was observed in all groups, with the HPFSC@PLA group maintaining the deepest staining. The quantitative results (Fig. 5B), obtained through ImageJ analysis, were consistent with the staining observations.

Calcium nodules are markers of late osteoblast differentiation, and Alizarin Red S (ARS) staining can predict this stage. As shown in Fig. 5C, compared to the control group, the HPFSC and HPFSC/3PLA groups exhibited fewer calcium nodules, while the HPFSC/5PLA and HPFSC@PLA groups displayed a significant number of stained calcium nodules. Quantitative analysis of the calcium nodules (Fig. 5D) corroborated the staining results, revealing that the ARS staining area in the HPFSC@PLA group reached 38.82 % ± 2.87 %.

### 3.7 Composite scaffold stimulates M2 polarization of macrophage

**Fig. 6:**
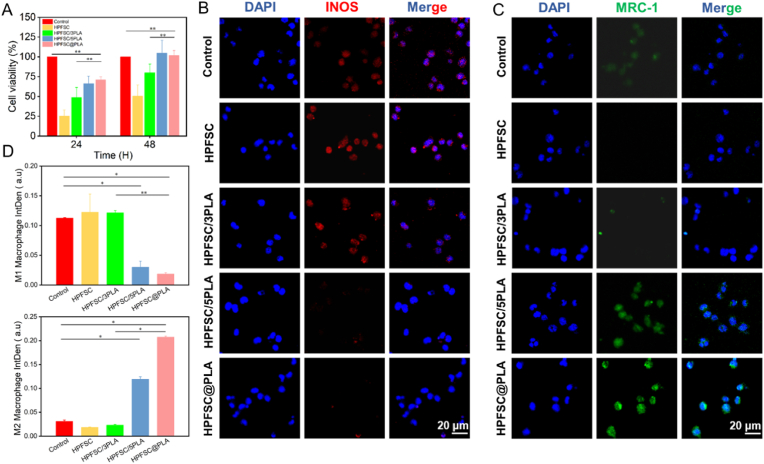
(A) Cell viability results of RAW267.4 cells; Macrophage phenotypic polarization immunofluorescence: (B) INOS expression level; (C) MRC-1 expression level; (D) Quantitative analysis of M1 and M2.

The immune response to implanted scaffold material is a crucial indicator of its efficacy. Research has shown that M1 macrophages can cause inflammation and impede bone tissue regeneration, while M2 macrophages promote bone tissue repair. This study investigated the effect of composite scaffolds on the transformation of M1 macrophages into M2 macrophages, starting with the induction of M0 macrophages into M1 macrophages. As shown in the fluorescence intensity results (Fig. 6B and C), the control and HPFSC groups exhibited strong red fluorescence, indicating high levels of M1 macrophages. The HPFSC/3PLA group showed slightly reduced red fluorescence. In contrast, the HPFSC/5PLA and HPFSC@PLA groups displayed minimal red fluorescence and strong green fluorescence, suggesting a higher presence of M2 macrophages. Quantitative analysis (Fig. 6D) confirmed these observations. Additionally, this study assessed the proliferation of RAW267.4 cells co-cultured with the composite materials for 48 h, mirroring the proliferation trend of MC3T3-E1 cells (Fig. 6A). PLA degradation produces hydroxyacetic acid and lactic acid, which are involved in carbohydrate metabolism. However, larger PLA materials can produce local acidic by-products during degradation, potentially leading to immune rejection and hindrance of bone regeneration. To address this, the FSC surface underwent minor PLA encapsulation treatment. The slow degradation rate of HPFSC/5PLA and HPFSC@PLA effectively prevented the rapid disintegration and accumulation of acidic by-products, thus reducing the immunogenicity and inflammatory reaction. This method shows that the hot-pressed composite scaffold coated with PLA shows low immunogenicity, effectively inhibits the inflammatory reaction after implantation, and promotes the transformation of M1-type macrophages into M2-type macrophages. Activation of JAK2/STAT1 by TLR4 pathway induced by LPS is an important mechanism of M1 polarization of macrophages. Toll-like receptors (TLRs), as type I transmembrane receptors, act on nuclear transcription factor-κ B (NF-κ B) and regulate the activation of macrophages. Different types of TLRs can recognize different signal molecules. LPS binds to TLR4 on the surface of macrophages, and then affects NF-κB and interferon regulatory factor 3 (IRF3), thus promoting the polarization of M1 macrophages. There are two subunits in NF-κB: p65 and p50, in which TLR2 drives the activation of p65 subunit to promote M1 polarization. When the p50 subunit is activated in the form of homodimer, it will promote the development of macrophages to M2 polarization.

As staining alone may not fully confirm the scaffold's ability to modulate inflammation, further cytokine release assays or gene expression level measurements will be conducted to provide more comprehensive evidence of the scaffold's impact on immune response in our future work.

### 3.8 In vivo femoral defect regeneration assessment

**Fig. 7:**
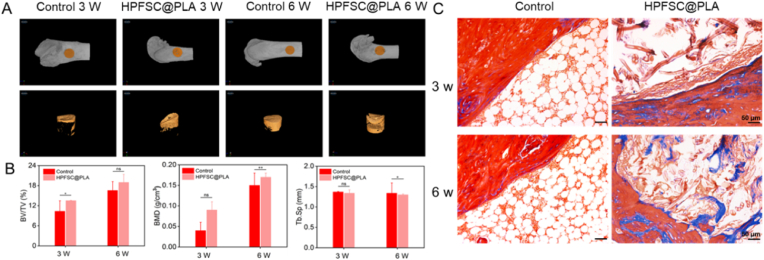
(A) Micro-CT images of 3 W and 6 W femoral defects; (B) Measurement of BV/TV, BMD, and Tb.sp through 3D reconstruction; (C) Masson staining images of defect site after implanting HPFSC@PLA composite materials for repair after 3 and 6 W.

To evaluate the in vivo bone defect repair capabilities of composite scaffolds, we constructed a rat femoral defect model with a diameter of 3 mm and a depth of 3 mm, implanting the HPFSC@PLA scaffold into the defect site. Micro-CT imaging (Fig. 7A) was utilized to observe bone regeneration at 3 and 6 weeks post-implantation, with quantitative analysis conducted on bone volume fraction (BV/TV), bone mineral density (BMD), and trabecular separation (Tb.sp). The results (Fig. 7B) indicated that at both 3 and 6 weeks, the BV/TV in the scaffold group was higher than in the control group, suggesting that the FSC/PLA composite scaffold significantly promoted femur repair. Additionally, the BMD and Tb.sp results showed that the quality of newly formed bone in the scaffold group was superior to that in the control group, with a lower risk of osteoporosis. Masson staining of the bone defect edges (Fig. 7C) further supported these findings. After 3 and 6 weeks of implantation, the HPFSC@PLA composite materials were visible within the defects, and the collagen fibers at the defect edges, stained blue, were more prominent compared to the control group. This evidence demonstrated that the scaffold continued to provide structural support and promoted new bone formation even after 6 weeks of implantation. Significantly, it cannot find local inflammatory cells in the HE staining pathological examination (Fig. 7C and S5) that showing good histocompatibility of the scaffold.

### 3.9 In vivo histological analysis of femoral defect regeneration

**Fig. 8:**
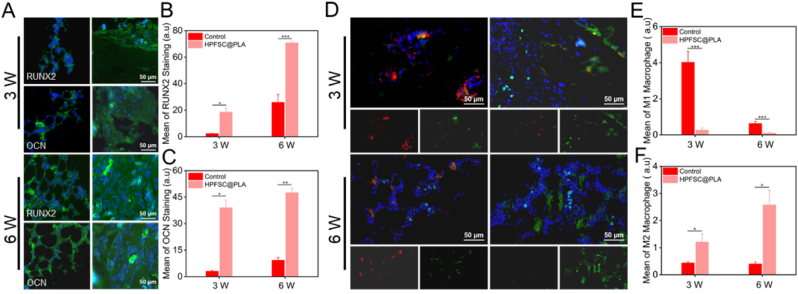
Immunofluorescence staining of macrophages and osteoblasts in the new bone zone. (A) Representative immunofluorescence images for osteogenic markers RUNX2 and OCN (green, osteogenic marker) after 3 and 6 weeks of operation. (B, C) Statistical data of mean fluorescence intensity of RUNX2 and OCN staining. (D) Representative immunofluorescence images for surface markers including iNOS (red, M1 phonotype), and MRC-1 (green, M2 phonotype) at 3 and 6 weeks operation. (E, F) Statistical data of the percentage of M1 and M2 macrophages.

RUNX2 is a crucial transcription factor integral to skeletal development and maturation. It regulates the differentiation, proliferation, and functionality of bone cells and is involved in various stages of bone tissue formation. Conversely, OCN is a protein specifically expressed in mature bone cells and serves as an indicator of mature osteoblast generation. As shown in Fig. 8A, after 3 and 6 weeks of repairing femoral defects with the implanted HPFSC@PLA composite material, both RUNX2 and OCN fluorescence intensities were lower in the control group compared to the HPFSC@PLA composite material group. The quantitative fluorescence intensity results across all groups (Fig. 8B and C) consistently demonstrated higher average fluorescence intensity in the HPFSC@PLA composite material group. These findings suggest that the HPFSC@PLA composite material more effectively promotes osteogenic differentiation and possesses superior osteogenic potential.

After bone injury, macrophages gradually transition from an M1 to an M2 phenotype, effectively suppressing local inflammatory reactions and providing a favorable microenvironment for osteogenic repair. This process is crucial for bone tissue regeneration and repair. In the third week of repairing femoral defects with the HPFSC@PLA composite material, as shown in Fig. 8D, fewer M1 macrophages with INOS-positive red fluorescence were observed in the HPFSC@PLA group compared to the control group, while more M2 macrophages with MRC-1-positive green fluorescence were present. The same trend was observed at the sixth week after implantation of the composite material for femoral defect repair, with even more M2 macrophages with MRC-1-positive green fluorescence in the sixth-week HPFSC@PLA group compared to the third-week HPFSC@PLA group. Quantitative results aligned with staining results, as shown in Fig. 8E and F. These findings indicate that the HPFSC@PLA composite material effectively stimulates the transition of M1 macrophages to an M2 phenotype and possesses immunomodulatory abilities on macrophage responses.

Based on our research findings, the HPFSC@PLA composite scaffold effectively stimulates the transition of M1 macrophages to the anti-inflammatory M2 phenotype, exhibiting strong immunomodulatory effects on macrophage responses while significantly enhancing bone regeneration. Three key mechanisms likely contribute to the scaffold’s inflammation modulation and osteogenesis promotion. Firstly, the FSC structure provides a unique microtopography that facilitates cell adhesion and influences cellular behavior. Previous studies have shown that specific surface features can modulate macrophage polarization, promoting the transition to an anti-inflammatory M2 phenotype, which is crucial for tissue regeneration and reduced inflammation. In addition, the mechanical properties of the FSC/PLA scaffold-such as its controlled stiffness and porosity-help create a favorable environment for osteogenesis by mimicking the mechanical characteristics of bone tissue. Then, silk fibroin in FSC itself has intrinsic bioactive properties that promote cellular interactions. Silk-based materials are known to support osteoblast adhesion, proliferation, and differentiation without the need for additional bioactive factors. Furthermore, silk fibroin has been shown to have immunomodulatory effects, promoting a balanced immune response by supporting the anti-inflammatory M2 macrophage polarization. The scaffold’s immunomodulatory and osteogenic effects arise from the synergistic effects of its structure design, mechanical properties, and the inherent bioactivity of silk fibroin, even in the absence of added bioactive factors.

## 4 Conclusion

In this study, we developed a controllable biodegradable composite scaffold composed of flat silkworm cocoon (FSC) and polylactic acid (PLA) to address bone healing in complex composite injuries. Our focus was on optimizing its structural design, mechanical properties, and the underlying mechanisms of osteogenesis. Results showed that the HPFSC@PLA composite scaffold exhibited excellent mechanical properties, biocompatibility, and an appropriate degradation rate, making it highly suitable for bone tissue engineering. It effectively promoted osteogenic differentiation, enhanced mineralization, and modulated macrophage polarization towards an anti-inflammatory M2 phenotype, contributing to reduced inflammation and improved bone regeneration. In vivo results from the rat femoral defect model confirmed its ability to support bone healing while minimizing postoperative complications. These findings demonstrate the potential of the HPFSC@PLA scaffold as an advanced material for bone repair applications.

## CRediT authorship contribution statement

**Jia Rui:** Writing – original draft. **Siyu Zhu:** Writing – original draft. **Xiang Xu:** Investigation. **Yi Wang:** Investigation. **Zulan Liu:** Writing – review & editing. **Guotao Cheng:** Writing – review & editing. **Dingpei Long:** Writing – review & editing. **Lan Cheng:** Writing – review & editing. **Fangyin Dai:** Writing – review & editing.

## Declaration of Competing Interest

The authors declare that they have no known competing financial interests or personal relationships that could have appeared to influence the work reported in this paper.

## Data Availability

Data will be made available on request.
