# Supplementary material for: High-performance silk/polylactic acid composite scaffold material with immunomodulation and osteogenesis function
Source: Mater Today Bio. 2024 Oct 28;29:101316. doi: 10.1016/j.mtbio.2024.101316 (PMC11570744; doi:10.1016/j.mtbio.2024.101316)
Supplement: Multimedia component 1 [file mmc1.docx]

**Hot pressing of polylactic acid/flat silkworm cocoon composite scaffolds for femoral defect regeneration**

**Supplemental Materials**


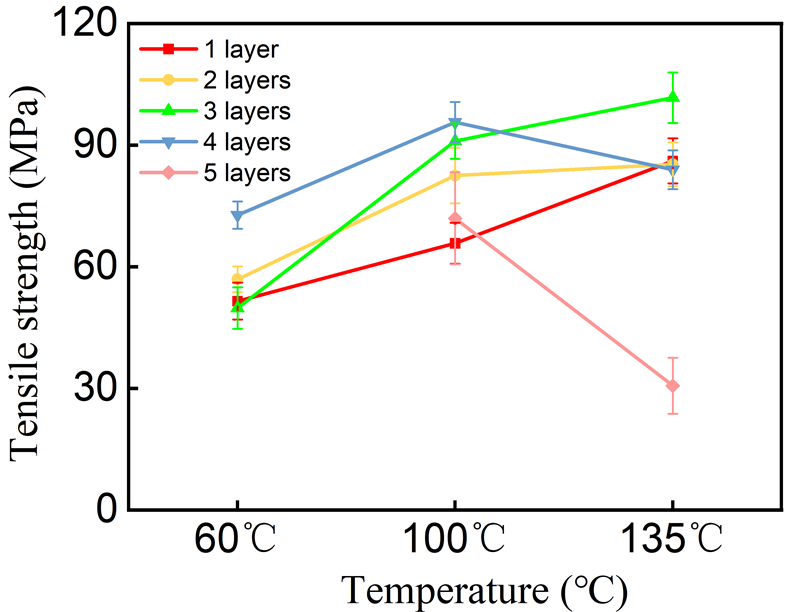


Fig. S1 Tensile testing after FSC hot pressing with different layers.


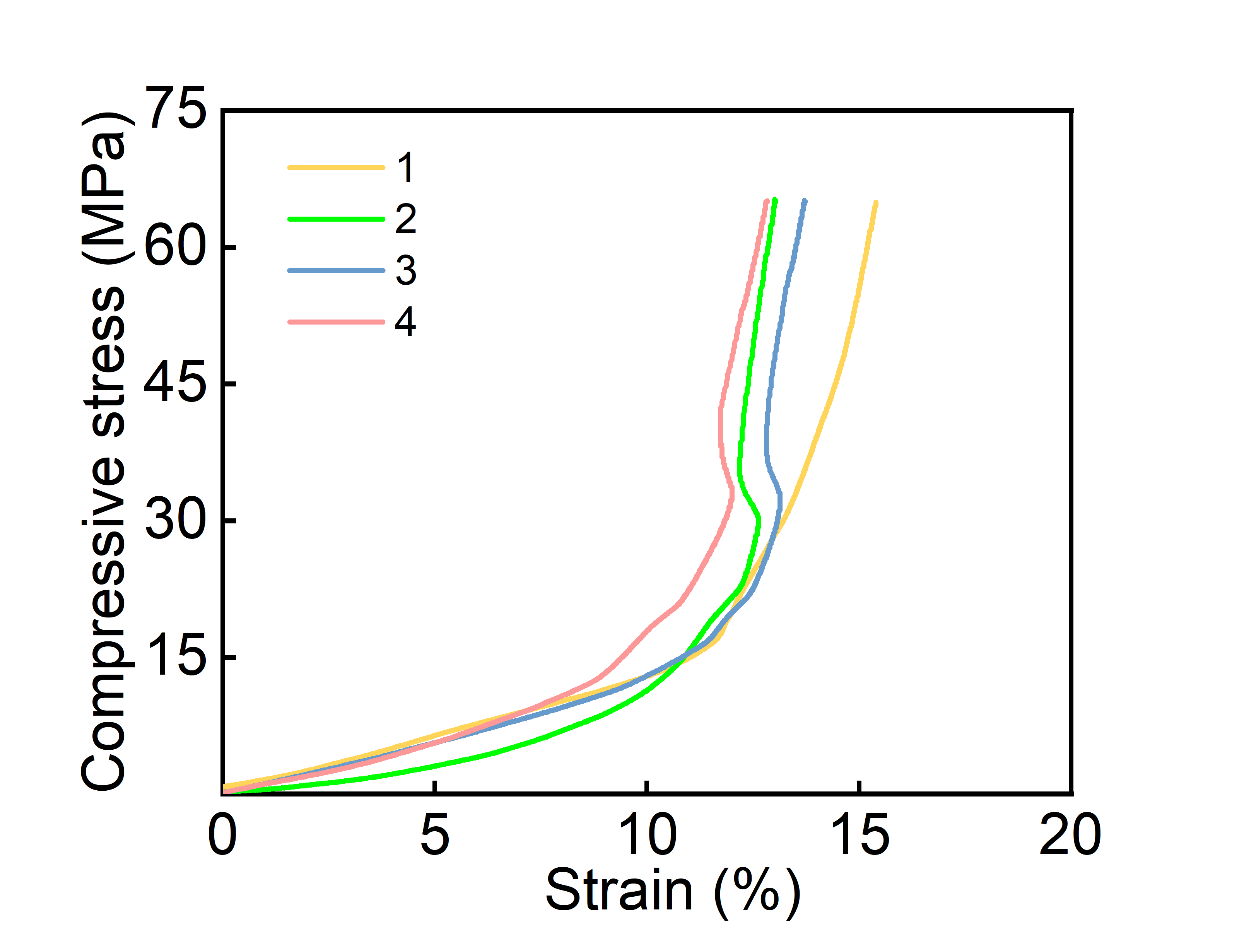


Fig. S2 Compression stress-strain curve of HPFSC-PLA-3 under 50N load.


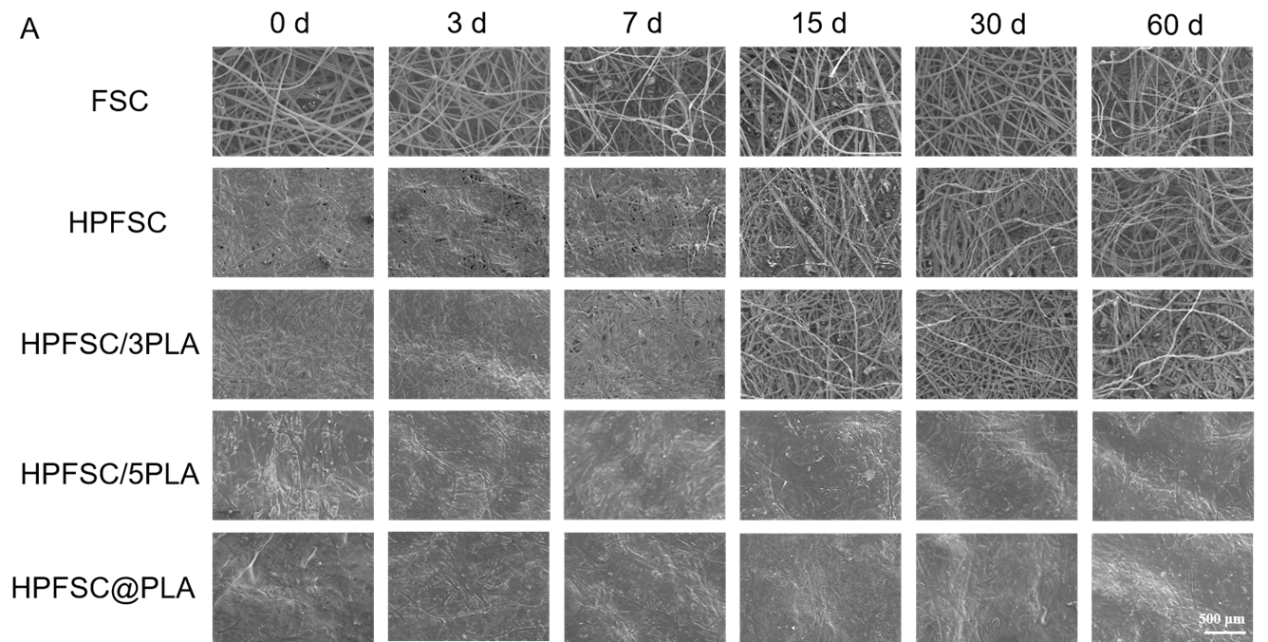


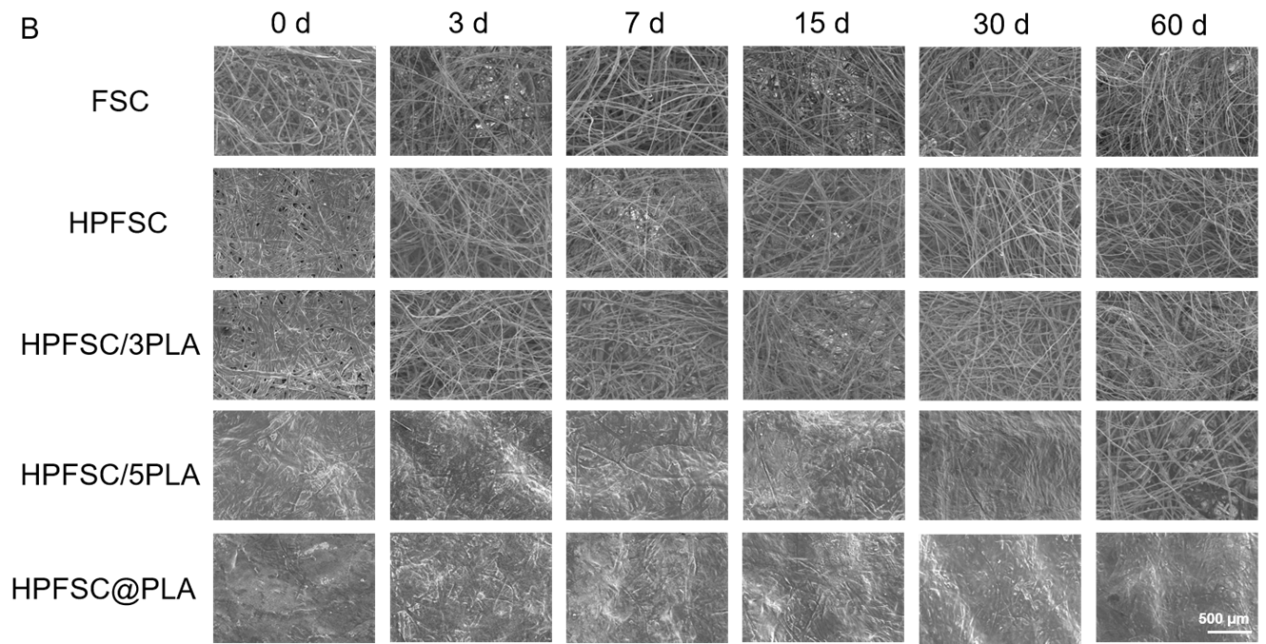


Fig. S3 SEM images of degradation of composite materials in PBS (A) and protease XIV PBS solution (B).


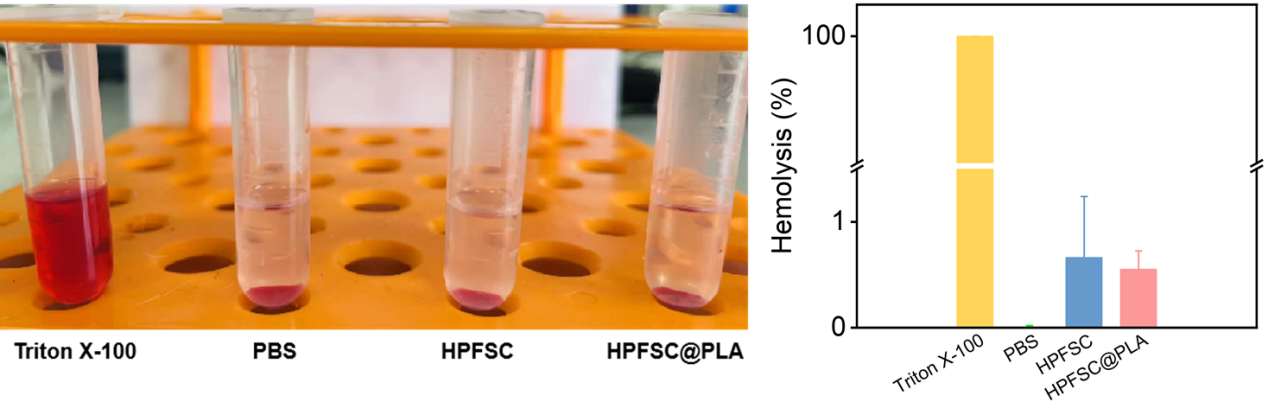


Fig. S4 Hemolysis of experimental groups treated with different materials.


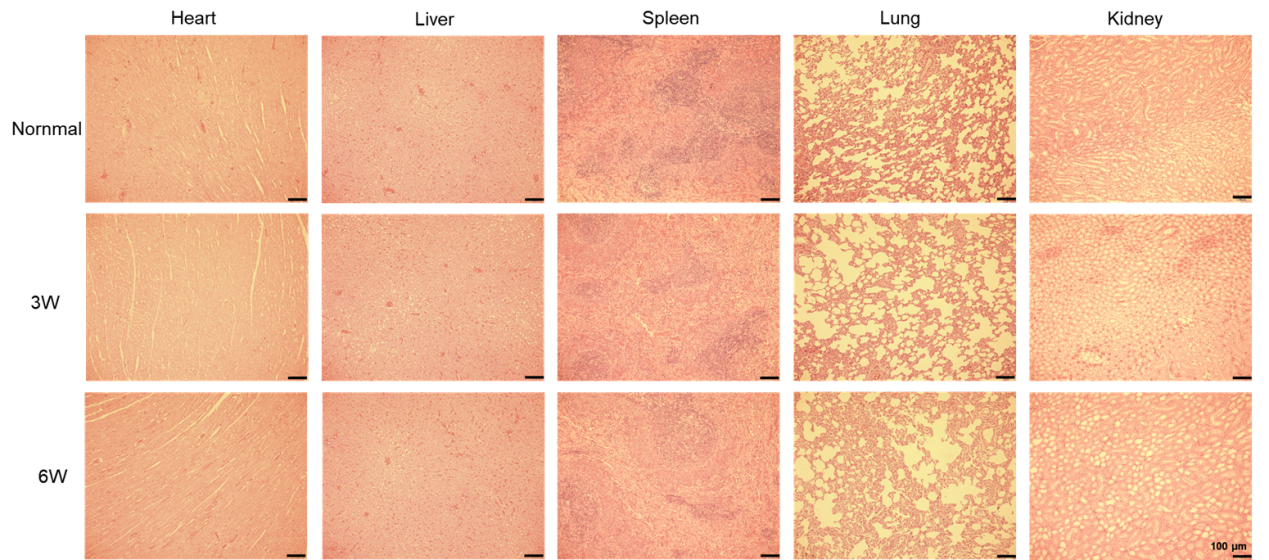


Fig. S5 H&E histopathological staining of five sections from SD rats 6 weeks after stent implantation.
